# Improved isolation of cadmium from paddy soil by novel technology based on pore water drainage with graphite-contained electro-kinetic geosynthetics

**DOI:** 10.1007/s11356-018-1664-4

**Published:** 2018-03-10

**Authors:** Xianqiang Tang, Qingyun Li, Zhenhua Wang, Yanping Hu, Yuan Hu, Miklas Scholz

**Affiliations:** 1 Basin Water Environmental Research Department, Changjiang River Scientific Research Institute, Wuhan, 430010 China; 2 Key Laboratory of Basin Water Resource and Eco-Environmental Science in Hubei Province, Wuhan, 430010 China; 3 Collaborative Innovation Center for Geo-Hazards and Eco-Environment in Three Gorges Area, Hubei Province, Yichang 443002 China; 4 Division of Water Resources Engineering, Faculty of Engineering, Lund University, PO Box 118, 22100 Lund, Sweden; 5 Department of Civil Engineering Science, School of Civil Engineering and the Built Environment, University of Johannesburg, Kingsway Campus, PO Box 524, Aukland Park, Johannesburg 2006 South Africa; 6 Civil Engineering Research Group, School of Computing, Science and Engineering, The University of Salford, Newton Building, Peel Park Campus, Salford, Greater Manchester M5 4WT UK

**Keywords:** Anode corrosion and cathode precipitation prevention, Atmospheric acid deposition, Electric energy consumption, Environmental remediation technology, Hazardous material, Cadmium, Paddy soil remediation, Water drainage

## Abstract

Novel soil remediation equipment based on electro-kinetic geosynthetics (EKG) was developed for in situ isolation of metals from paddy soil. Two mutually independent field plot experiments A and B (with and without electric current applied) were conducted. After saturation using ferric chloride (FeCl_3_) and calcium chloride (CaCl_2_), soil water drainage capacity, soil cadmium (Cd) removal performance, energy consumption as well as soil residual of iron (Fe) and chloride (Cl) were assessed. Cadmium dissolved in the soil matrix and resulted in a 100% increase of diethylenetriamine-pentaacetic acid (DTPA) extracted phyto-available Cd. The total soil Cd content reductions were 15.20% and 26.58% for groups A and B, respectively, and electric field applications resulted in a 74.87% increase of soil total Cd removal. The electric energy consumption was only 2.17 kWh/m^3^ for group B. Drainage by gravity contributed to > 90% of the overall soil dewatering capacity. Compared to conventional electro-kinetic technology, excellent and fast soil water drainage resulted in negligible hydrogen ion (H^+^) and hydroxide ion (OH^−^) accumulation at nearby electrode zones, which addressed the challenge of anode corrosion and cathode precipitation of soil metals. External addition of FeCl_3_ and CaCl_2_ caused soil Fe and Cl residuals and led to 4.33–7.59% and 139–172% acceptable augments in soil total Fe and Cl content, correspondingly, if compared to original untreated soils. Therefore, the novel soil remediation equipment developed based on EKG can be regarded as a promising new in situ technology for thoroughly isolating metals from large-scale paddy soil fields.

**Electronic supplementary material:**

The online version of this article (10.1007/s11356-018-1664-4) contains supplementary material, which is available to authorized users.

## Introduction

Electro-kinetic remediation is used to isolate metals such as Cd from polluted soil (Suer et al. 2003). When a low voltage of direct current or a low potential gradient of an electric field is applied, metals including Cd in soil will migrate to electrode chambers by electro-migration, electro-osmotic flow and electrophoresis. In the electric field, positive Cd^2+^ and cations migrate to the cathode, while negative chlorides as well as anions migrate to the anode.

Accumulated Cd near the cathode area can be removed though electroplating, adsorption onto the electrode, precipitation or co-precipitation at the electrode, pumping water near the electrode and complexation with ion exchange resins (Tang et al. 2016).

In low buffering soils, an electrolysis reaction results in the soil pH to decrease to 2–3 near the anode and to increase to 8–12 near the cathode during the electro-kinetic remediation process (Giannis and Gidarakos 2005). Therefore, soil acidification may occur in the anode area, preventing Cd from being adsorbed to the soil particles or being precipitated as hydroxides, oxyhydroxides, etc. Conversely, soil alkalisation may occur in the cathode area, which causes the precipitation of Cd inside the soil matrix. Besides the challenge of pH control, the majority of the electro-kinetic remediation studies were conducted with sieved artificially contaminated soil, and experimental Cd contents are commonly hundred times higher than those in naturally contaminated paddy soil (Chen et al. 2015; Cameselle and Pena 2016).

To date, most of the electro-kinetic remediation studies were conducted with sieved artificially contaminated soil, and the experimental Cd content is often hundred times higher than those in naturally contaminated farmland soil. Moreover, all bench-scale experiments were conducted in the laboratory within a soil column of usually less than 1 m in terms of length, and graphite was the most commonly used electrode material to remediate Cd contamination (Tang et al. 2016).

Current electro-kinetic remediation equipment usually consists of parts, such as anode, cathode, anode chamber, cathode chamber, ion exchange membrane, current source and pump (Chen et al. 2015). An indispensable electrode chamber, unavoidable polarisation, corrosion of anode and hard-to-separate metals from soils greatly limit the field application of electro-kinetic technology for detoxification of Cd-contaminated paddy soil.

In the paddy soil ecosystem, soil particles, pore water and crop biomass are the major environmental media for toxic Cd storage. Root uptake and transfer of dissolved Cd in soil water can be regarded as the most important contributors to Cd contamination in grain (Rafiq et al. 2014; Hu et al. 2015). As the strength, volume and movement of dissolved Cd in paddy soil are directly related to soil pore water, pore water movement and drainage are highly beneficial. Therefore, dissolving and releasing of Cd from soil particles to soil water, and then to maximise the drainage of Cd-enriched soil pore water is the most promising and fundamental solution for remediating Cd-contaminated paddy soil and guarantee the safe production of grain in the future.

Compared to traditional electro-kinetic remediation, electro-kinetic geosynthetics (EKG) involves the process of electro-osmosis, electrophoresis and associated electro-kinetic functions such as electrolysis with the traditional functions of geosynthetics comprising drainage and filtration. Therefore, EKG technology shows promise in remediating Cd-contaminated paddy soil, where electroosmosis can achieve flow rates of up to four orders of magnitude greater than hydraulic flow.

The EKG comprises conducting elements coated onto corrosion-resistant material. This patented design has overcome the challenge of electrode corrosion and the removal of water through drainage and filtration. The soil water can be quickly drained via gravity by water drainage grooves designed within geosynthetics without the presence of an electric current (Glendinning et al. 2007). Soil pore water can be drained rapidly through a water drainage groove or a guiding gutter designed into the EKG sheet. When an electric field with a low direct current voltage is applied, besides gravity drainage, pore water is directionally driven from the anode to the cathode (Jones et al. 2008). However, the lack of specialised equipment suitable for pore water collection, separation, storage and drainage hinders EKG application in the remediation of Cd-contaminated paddy soil.

**Fig. 1:**
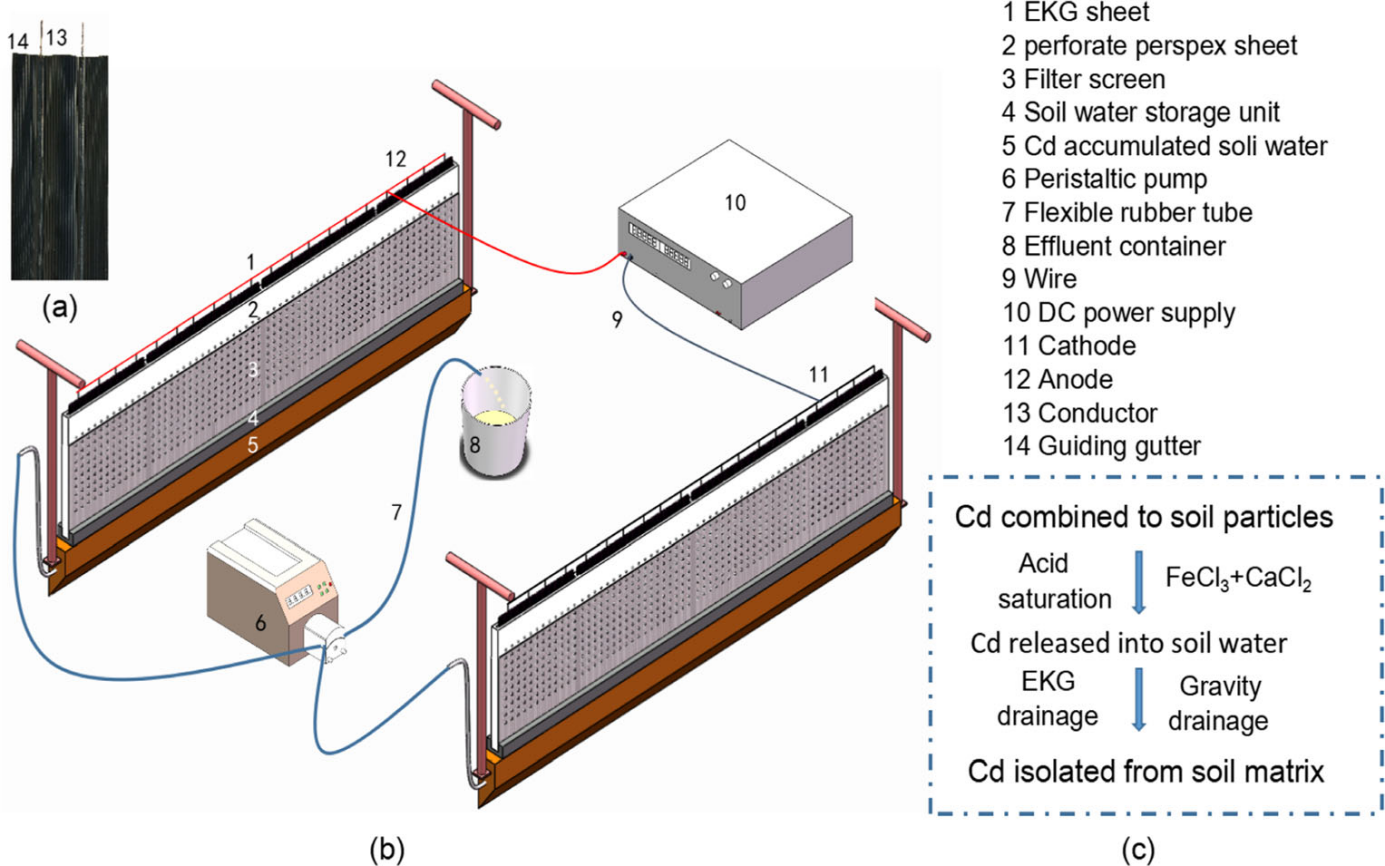
Schematic of (**a**) the electro-kinetic geosynthetics (EKG) structure; (**b**) novel EKG equipment component with direct current (DC) power supply; and (**c**) schematic for isolating cadmium (Cd) from paddy soil using ferric chloride (FeCl_3_) and calcium chloride (CaCl_2_)

In order to efficiently separate Cd from paddy soil particles via draining soil water, the novel technology was developed based on EKG (Fig. 1). The new equipment uses commercially obtained EKG as electrode, which adopts graphite for electric conduction. Moreover, soil water can be quickly drained with the novel equipment after Cd is released from paddy soil particles via dissolution by acid reagents, and the soil phyto-available Cd can be isolated with minimal volume of soil water drainage. For guiding actual remediation practices, the EKG equipment was used to conduct an in situ field plot experiment, and aimed to (a) determine the soil dewatering efficiency for the EKG equipment with and without electric current; (b) investigate the polarisation effect during the electro-kinetic remediation process with the application of the EKG equipment; (c) evaluate the improvement of soil Cd removal by using the EKG device; and (d) assess the soil residual of the acid reagent introduced to facilitate the Cd removal via electro-kinetic remediation.

## Materials and methods

### Experimental site and soil properties

The in situ electro-kinetic remediation experiments were conducted in Beishan Town (North 28°26′38″; East 113°03′50″), Changsha City, Hunan Province, China. The paddy soils in this province are contaminated by metals including Cd. Moreover, Changsha City is subjected to sulphuric acid-type acid rain pollution (Wang et al. 2010), which increases the mobility and phyto-availability of Cd within paddy soil, which causes grain contamination.

For the paddy soil in Beishan Town, the top 10 cm were used for electro-kinetic remediation. Local paddy soils have been contaminated for a long time. The estimated average background level of soil Cd content is 0.097 mg/kg (Tang et al. 2016). Long-term irrigation with Cd-enriched river water resulted in considerable soil Cd contamination of 0.83–1.04 mg/kg, which represents a moderate to high contamination level with respect to the Environmental Quality Standard for Soils (GB15618-2008) in China (MEP 2008). Due to atmospheric acid deposition, the paddy soil in Beishan Town was weakly acidic and was comprised of sandy-loam.

### Novel experimental equipment

The electro-kinetic equipment used for isolating metals from paddy soil consisted of three main units: electro-kinetic remediation, soil water storage and auxiliary (Fig. 1). The electro-kinetic remediation unit is a sandwich structure with a graphite coated EKG sheet as the middle layer and two perforate Perspex sheets as the external layers, which were used to fix and protect the flexible EKG sheet. A filter screen covers the external surface of the perforate Perspex sheet to prevent clogging with soil particles. A vertical guiding gutter within the EKG sheet facilitates soil water collection and subsequent migration to the soil water storage unit. The perforate Perspex sheet, graphite coated EKG sheet and filter screen had the same length (1.00 m) and height (0.30 m), but different thicknesses: 3, 1 and 0.5 mm, respectively.

When a direct current electric was applied, soil water and soluble Cd gradually migrated to the nearby areas of the cathode, and then entered into the cathode’s soil water storage unit via gravity and electro-migration. Some soluble Cd and soil water nearby the anode could also directly move into the anode’s soil water storage unit.

### Experimental set-up and operation

In May 2017, the performance of Cd removal with the new EKG equipment was investigated under real contamination conditions. Four field plots were categorised into two groups (A and B). Each group had two identical plots with the same dimensions of 1.50 m in length, 0.86 m in width and 0.25 m in soil depth. Each plot was sealed with a waterproof separator to the depth of 0.25 m (Supplementary Material Fig. S1). The detailed experimental design can be found in the Supplementary Material Table S1. For group A, no electric field was applied to test for Cd removal using soil water drainage by gravity. In comparison, a direct current electric field of 100 V was applied for group B to test the combined effect of electro-migration and gravity drainage, which used 2 V/cm as the voltage gradient that was optimised based on previous electro-kinetic remediation practices involving traditional electrodes such as column graphite (Probstein and Hicks 1993).

The initial average water content of the four plots was 38%. For each plot, 47.5 L saturated solution (pH of 2.31) containing 0.03 mol/L FeCl_3_ and 0.03 mol/L CaCl_2_ was fully and evenly mixed with the top 10 cm cultivated soil layer to increase the mobility and bioavailability of Cd within the soil. After saturation for 24 h, approximately 0.6 cm of overlying water was observed on the soil surface, and the actual volume ratio of solid to liquid was 6.80. Thereafter, the liquid stored in the water storage units was drained by a peristaltic pump for 2 h, which then resulted in no obvious overlying water on the soil surface for groups A and B. This first drainage may be regarded as overlying water. Subsequently, the electric field was applied for group B. Therefore, groups A and B both operated for 32 h, but a total of 20 h was used for electro-kinetic remediation.

### Sampling and analysis

During the experimental period, water drainage was conducted at 1, 2, 4, 8, 12, 24, 28 and 32 h for groups A and B. The overlying water and the soil water were tested for pH, Cd, Fe and Cl. The pH values were measured via a portable HANNA pH meter. After three acids (hydrofluoric, nitric and perchloric) digestion, Cd and Fe were identified by atomic absorption spectrophotometry (AAS; AA-400, PerkinElmer). Chlorine was analysed with Shim-pack ICA1 (Shimadzu DGU-12A) using 2.5-mM phthalic acid combined with 2.4-mM tris-(hydroxymethyl) aminomethane (Lin 2002). The electric current for group B was recorded.

For groups A and B, the electrode space was 0.5 m. The variations in soil Cd contents before and after remediation, and the occurrence of soil Cd leakage from the top soil layer to the deep soil layer after 24 h acid (FeCl_3_) saturation were assessed. The soil columns were subdivided into about 0–5, 5–10, 15–20 and 20–25 cm samples. Soil samples were air-dried, screened and digested. The phytoavailable Cd fraction was extracted using DTPA according to Lindsay and Norvell (1978) and specified further by Baldantoni et al. (2009). For determining Cl, 0.25-g sieved soil samples were extracted using 25 mL of deionised water. About 438 g were centrifuged at 3500 rpm for 3 min. The supernatant was analysed using the above procedures (Lindsay and Norvell 1978). The profile soil pH, total Cd, phytoavailable Cd, total Fe and Cl content were calculated as the average of three columns. Furthermore, the electrical energy consumption was calculated according to Yuan et al. (2016).

All analytical instruments passed national measurement examinations, and the recovery rates of Cd detection via AAS analysis were between 95% and 110%. Moreover, reference materials and standard reagents were used for quality control purposes. All experiments and measurements were conducted as duplicates, and averages were calculated. Moreover, regardless of group A or B, Cd, Fe and Cl concentrations for the three soil columns were averaged for each subdivided layer and reported as mean values.

## Results

### Dewatering performance

For groups A and B, nearly 10.50 and 10.82 L overlying water was drained within 2 h, respectively. As shown in Supplementary Material Fig. S2, 7.87 and 10.22 L of total soil water were drained for groups A and B after 32 h of operation, respectively. Moreover, approximately 75% of the total soil water drainage occurred within the first 12 h. Thereafter, the soil dewatering velocity decreased.

Without the presence of an electrical field, curves of soil water drainage for the anode and cathode had similar distributions. The introduction of electro-kinetic practices greatly changed the original soil dewatering status. About 6.61 and 3.61 L of soil water had been drained from the cathode and anode (Supplementary Material Fig. S2), respectively. The electric field promoted the migration of soil water from anode to cathode. The electric energy consumption decreased as the treatment duration increased. The measured current intensity gradually decreased from 1.02 to 0.22A (Supplementary Material Fig. S3). This means that more soil water was drained, and more power input was required. During the whole experimental period, the calculated energy consumption was 2.17 kWh/m^3^ when considering the treated soil volume of group B.

### Drainage water quality

**Fig. 2:**
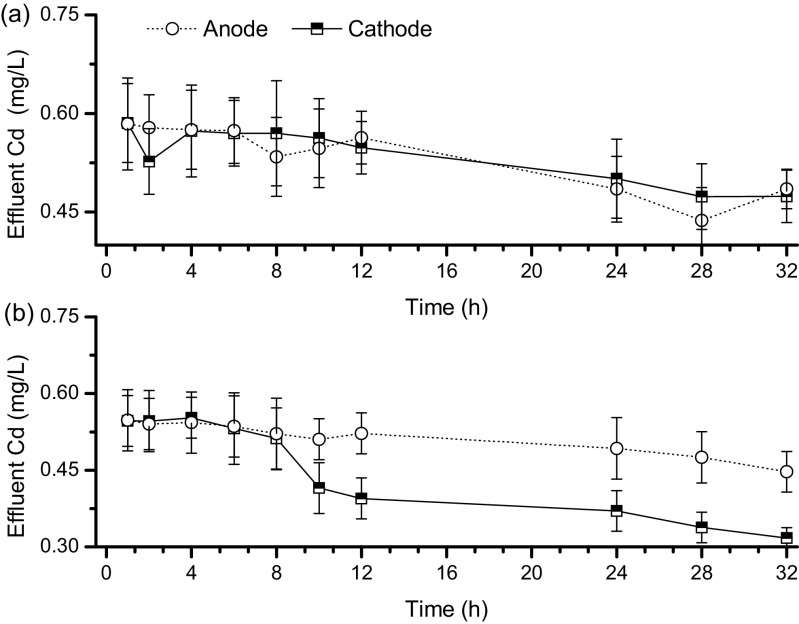
Variations in anode and cathode effluent cadmium (Cd) concentrations for (**a**) group A and (**b**) group B

Effluent Cd, Fe and Cl concentrations were measured during the treatment period. As shown in Fig. 2 and Supplementary Materials Figs. S3 and S4, negligible differences had been observed for Cd, Fe and Cl concentration curves between anode and cathode when the soil water was only drained by gravity. In general, the effluent Cd, Fe and Cl concentrations decreased as the experimental duration increased, regardless of using an electric field or not. This implies that the migration capability of soil-dissolved Cd, Fe and Cl decreases with a decline in soil water content. The presence of an electric field contributed to the change in effluent Cd, Fe and Cl concentrations for group B. As shown in Fig. 2 as well as Supplementary Materials Figs. S3 and S4, much higher Cd, Fe and Cl loads were detected from the anode effluent when compared to the corresponding values obtained from the cathode. For group B, the effluent Cd concentrations were 0.45 and 0.32 mg/L after 32 h of anode and cathode operation, respectively.

As shown in Supplementary Material Fig. S5, drainage by gravity caused similar patterns for anode and cathode effluent pH curves. The recorded pH slowly increased from 2.47 to 2.92. The anode effluent pH slightly decreased from 2.13 to 1.69, while the cathode effluent pH gradually increased from 2.33 to 2.75. Different effluent pH values caused a significant (*p* < 0.05) difference in iron ion valence, which resulted in green and orange colours that were apparent for both anode and cathode effluents (Supplementary Material Fig. S1). This indicated that Fe^2+^ and Fe^3+^ dominated anode and cathode effluent in this order. Furthermore, Supplementary Material Fig. S6 shows the variations in anode and cathode effluent Cl concentrations.

### Soil Cd content variation

The cultivated top layer of soil saturated with 0.03 mol/L FeCl_3_ and 0.03 mol/L CaCl_2_ increased the active Cd content. The DTPA-extracted Cd content in the top 10 cm of the soil layer increased to nearly 0.55 mg/kg after 24 h of saturation, when compared to 0.30 mg/kg of untreated soil. Augmentation of dissolved and mobilised DTPA-extracted Cd facilitated its subsequent isolation and removal via soil water drainage. The overlying water and the soil water drainage caused 10.66 mg and 12.33 mg Cd removal from the soil matrix for groups A and B, respectively (Table 1). The application of an electric field led to a 42.35% increase in Cd removal by soil water drainage.

**Table 1:** Soil cadmium (Cd), iron (Fe) and chlorine (Cl) removal via water drainage during electro-kinetic treatment

Parameter	Group A		Group B
	Anode	Cathode	Anode
Total water drained (L)			
Overlying water^a^	10.50 ± 0.73		10.82 ± 0.68
Soil water	4.22 ± 0.14	3.65 ± 0.21	6.61 ± 0.32
Total Cd removal (g)			
Overlying water	6.41 ± 0.35		6.28 ± 0.19
Soil water	2.28 ± 0.12	1.97 ± 0.20	4.19 ± 0.13
Total Fe removal (g)			
Overlying water	34.23 ± 2.46		31.70 ± 1.92
Soil water	10.07 ± 1.37	8.04 ± 0.91	23.97 ± 1.26
Total Cl removal (g)			
Overlying water	117.04 ± 5.26		120.86 ± 3.78
Soil water	30.18 ± 3.15	26.80 ± 2.78	52.71 ± 4.32
Energy consumption (kWh)^b^	Not applicable		0.70 ± 0.12

^a^There was no difference regarding the overlying water drainage between anode and cathode before the electro-kinetic remediation experiments; therefore, the overlying waters drained from the anode and cathode had been collected together

^b^Group A was not influenced by electro-kinetic operation. The power consumption for soil water drainage was negligible

**Fig. 3:**
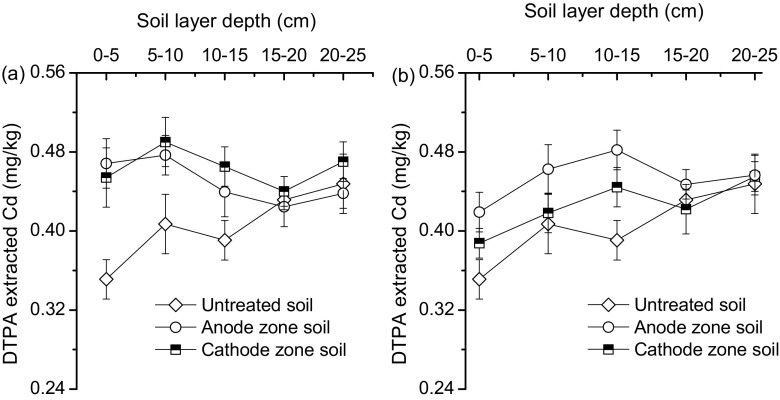
Change in profile distribution of soil diethylenetriamine-pentaacetic acid (DTPA)-extracted cadmium (Cd) content for (**a**) group A and (**b**) group B with untreated original soil as reference

After 32 h of operation, the DTPA-extracted Cd content within the top cultivated soil layer showed a significant decline, if compared to the concentration of 0.55 mg/kg caused after saturation. When the soil pH value was adjusted to its initial level of 5.2 through 1.0 kg/m^2^ lime neutralisation after the experiment, the soil DTPA-Cd content sharply decreased to 0.25 mg/kg (data not shown) due to in situ stabilisation and immobilisation, which created a favourable condition for crop cultivation. The top 10 cm of cultivated soil DTPA-extracted Cd content decreased to a concentration range between 0.38 and 0.49 mg/kg (Fig. 3). Regardless of the electric field application, the DTPT-extracted Cd content within the top 15 cm of cultivated soil for groups A and B were significantly (*p* < 0.05) higher than the corresponding values measured for the untreated soil (Fig. 3).

**Fig. 4:**
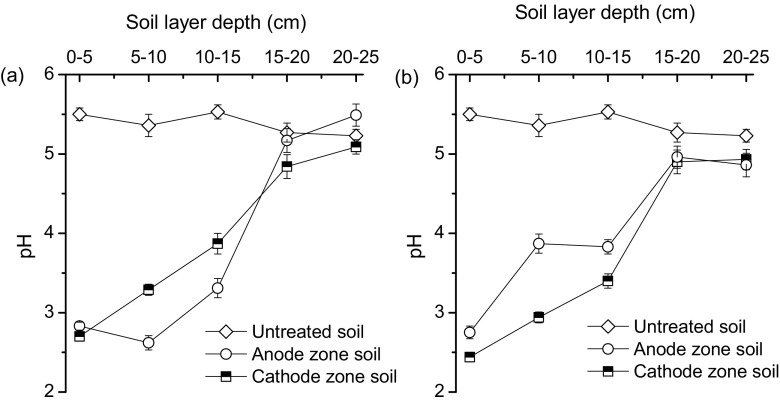
Change in profile distribution of soil pH values for groups A (**a**) and B (**b**) with untreated original soil as reference

The introduction of FeCl_3_ reduced the soil pH, so that the pH values of the top 15 cm of soil ranged from 2.42 to 3.83, which was significantly lower than approximately 5.50 for the identical untreated soil layers (Fig. 4). Acid soil environments enhanced the dissolvability and mobility of soil combined Cd, and thus resulted in a relatively high DTPA-extracted Cd content. There was no obvious difference in soil DTPA-extracted Cd content for the soil layer (15 to 25 cm) between treated groups and the untreated control (Fig. 3), which demonstrated Cd leakage from the top cultivated soil layer to the undisturbed deep soil layer.

Compared to group A, the presence of an electric current changed the soil DTPA-extracted Cd spatial distribution. The anode zone soil DTPA-extracted Cd concentration was higher than the measured one in the cathode zone (Fig. 3). The average DTPA-extracted Cd concentrations in the top 15 cm of the soil were 0.45 and 0.41 mg/kg for the anode and cathode zone in group B, respectively.

**Fig. 5:**
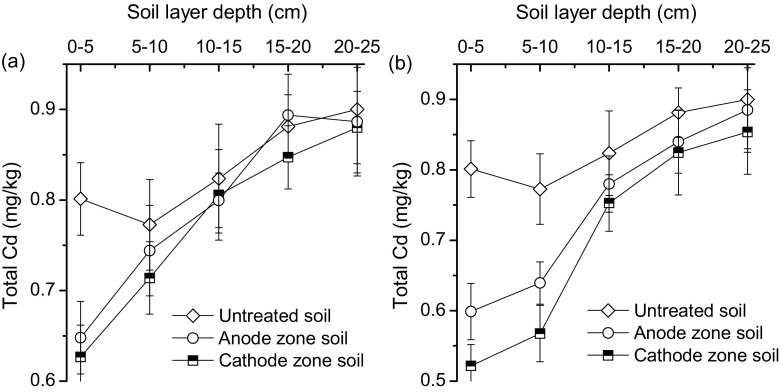
Change in profile distribution of soil cadmium (Cd) content for (**a**) group A and (**b**) group B with untreated original soil as reference

The removal of soil water drainage resulted in a reduction of DTPA-extracted Cd. This led to a direct decline in the total Cd content in the top 10 cm of soil. Compared to untreated soil, the average total Cd content within the top 10 cm reduced from 0.79 to 0.67 mg/kg and 0.79 to 0.58 mg/kg (Fig. 5), which corresponded to 15.20% and 26.58% removal for groups A and B, respectively. The application of an electric current caused 74.87% improvement of soil total Cd removal, when gravity drainage was used.

### Soil Fe and Cl residuals

Soil water drainage can remove the majority of added Fe and Cl. As shown in Supplementary Material Table S2, the overlying water and soil water discharge can remove nearly 52.34 and 63.48 g Fe, and 165.50 and 203.91 g Cl for groups A and B, correspondingly. When balanced to the initial added loads, there are 20 to 35% residuals for Fe and Cl, respectively. Soil Fe and Cl residual rates for group B were 15% lower, if compared to the corresponding values obtained for group A (Supplementary Material Table S2). When referred to untreated soil, the residual of Fe was negligible, and only caused a slight augmentation in Fe content within the top 15 cm of soil (Supplementary Material Fig. S7). However, the residual of Cl was significant. Accumulated Cl caused a relatively high Cl content in the top 10 cm (particular within the top 5 cm). When compared to untreated soil background values, the Cl residual led to soil Cl concentrations that were between 2 and 4 times higher (Supplementary Material Fig. S8).

## Discussion

### Mechanisms of Cd removal

Previous research indicated that the extraction of Cd can be > 60%, if the soil pH is < 3 (Giannis et al. 2010). Therefore, acids, chelating agents and surfactants may be applied as saturation solutions for soils before electro-kinetic treatment to create an acid environment (Hahladakis et al. 2014). In this study, a solution comprising 0.03 mol/L FeCl_3_ and 0.03 mol/L CaCl_2_ with an initial pH of 2.31 was used to saturate the soil for 24 h. The introduction of Fe^3+^ and Ca^2+^ competitively exchanged Cd^2+^ that was previously combined with hydroxides and oxyhydroxides. The DTPA-extracted phyto-available Cd increased to 0.55 mg/kg after 24 h of saturation, when compared to 0.35 mg/kg for the original soil, which provided excellent preconditions for the subsequent isolation of dissolved soil-based Cd by electro-kinetic remediation.

The presence of EKG favoured the migration and removal of dissolved soil-based Cd through soil water drainage, which greatly facilitated the isolation of Cd from the soil matrix. Insignificant increases or decreases in the top 15 cm of the soil pH near the anode and cathode were recorded for group B after 32 h of treatment (Fig. 4). No obvious H^+^ and OH^−^ accumulation occurred during the process of electro-kinetic remediation. The reason was that the majority of the H^+^ and OH^−^ ions were generated through electro-osmosis, and easily drained via vertical gutters in the EKG sheet.

The EKG equipment has created short pathways for soil water as well as dissolved Cd to flow out of a low permeability soil matrix via processes such as electro-migration and electro-osmosis. Drainage promoted dissolved Cd removal. The soil dewatering performance showed a similar pattern to that of the soil total Cd removal (Supplementary Material Fig. S2 and Fig. 2). The more soil water was drained, the greater was the reduction in soil total Cd. After 32 h of operation, groups A and B drained 18.37 and 21.04 L, which resulted in 10.66 and 12.33 mg removal of soil dissolved Cd, respectively (Table 1). Moreover, the application of the electric current only resulted in 29.86% increase in soil water dewatering performance for group B, if compared to group A. Drainage by gravity performed much better than electro-osmosis.

The electric current intensity decreased with the extension of operation time (Supplementary Material Fig. S3). Repeated soil water drainage activities reduced the soil water ionic strength, and thus increased the electric resistance. A similar phenomenon can also be found in other electro-kinetic remediation techniques involving the use of EKG (Jones et al. 2011; Lamont-Black et al. 2015).

Although the top 10 cm of paddy soil experienced 24-h saturation with FeCl_3_ and CaCl_2_, no obvious dissolved Cd leakage was detected after 32 h of electro-kinetic remediation. There was no difference in total Cd and DTPA-extracted Cd content in the soil layer below 20 cm between groups A and B (Figs. 3 and 4), which can be attributed to the following two aspects: (1) The low permeability of the long-term undisturbed deep soil layer hindered the infiltration of dissolved Cd; and (2) The EKG equipment created a short pathway for fast drainage of collected soil water from the top 10 cm of soil, and reduced the chance of soil water vertical leakage.

### Impacts of Fe and Cl residuals

The introduction of FeCl_3_ and CaCl_2_ assisted the electro-kinetic remediation process, but caused unavoidable residual of Fe, Ca and Cl. Limestone is commonly added to adjust the acid soil environment in Changsha City, so the moderate residual of Ca in the present study was beneficial. Electro-kinetic remediation with EKG equipment can remove Fe^3+^ and Cl^−^. Soil water drainage caused 52.34 and 63.48 g removal in Fe^3+^ as well as 165.50 and 203.91 g removal in Cl for groups A and B (Supplementary Material Fig. S6), which corresponds to 20.45–34.41% in Fe and 19.37–34.56% in Cl residual of the total input, correspondingly.

Soil water migrates from the anode to the cathode when an electric field is applied (Jones et al. 2011). Thus, more removal of Fe^3+^ and Cl^−^ from the cathode compared to the anode can be noticed. Despite of 65–80% removal of Fe^3+^ and Cl^−^, only 4.33–7.59% and 139–172% augmentation in soil Fe and Cl content can be achieved, if compared to the untreated soil. The addition of low concentration of FeCl_3_ and CaCl_2_ resulted in acceptable Fe^3+^ and Cl^−^ residuals.

Previous research indicated that more than 40% of electric energy was consumed during Fe removal (Yuan et al. 2017). However, moderate residual of Fe^3+^ was useful to hinder the uptake of soil phyto-available Cd during the rice growing period and enhance the nutrient uptake and avoid heavy metal toxicity (Fu et al. 2010). Furthermore, residual of Fe and Cl can improve migration of metals under acid environments (Zhou et al. 2005; Iannelli et al. 2015), which can be used for extended or follow-up electro-kinetic treatments.

### Cost-efficiency analysis

The EKG equipment exhibited nearly 30% removal of soil total Cd content after 32 h of in situ field plot remediation, though the treatment efficiency was somewhat lower than that reported range from 58 to 98% obtained in laboratory experiments (Almeira et al. 2012; Missaoui et al. 2016; Yuan et al. 2016). Several reasons resulted in the relative low soil Cd removal in the present study: (1) Small quantities of sieved artificially contaminated soil particles in common electro-kinetic remediation practices, and a high concentration of externally added Cd and uniform soil properties result in a high removal efficiency; (2) Conventional electro-kinetic remediation usually lasts between days and years (Virkutyte et al. 2002; Missaoui et al. 2016); (3) During the conventional electro-kinetic remediation process, more than 60% of the metals precipitate in the catholyte or accumulated in nearby areas of the cathode (Almeira et al. 2012), which may not reflect the actual removal.

For this preliminary trail, however, in situ electro-kinetic remediation of Cd-contaminated paddy soil was relatively high (approximately 30% reduction in total Cd). In order to further remove the soil DTPA-Cd, repeated electro-kinetic remediation with the novel EKG equipment can be conducted to gradually reduce the remaining soil Cd during the non-growing season, and then subsequently use lime to stabilise and immobilise the remaining Cd during the growing season, and guarantee crop safety during cultivation (Tang et al. 2016).

The EKG equipment exhibited advantages in terms of energy saving: < 2.17 kWh/m^3^ electricity was consumed by group B (Supplementary Material Fig. S3 and Table 1) to migrate and isolate Cd, Fe and Cl from the soil matrix via pore water drainage. Electric energy consumption for conventional electro-kinetic remediation of metal-contaminated soils varies from 38 to 1264 kWh/m^3^ (Virkutyte et al. 2002; Cang and Zhou 2011; Yuan et al. 2016, 2017). The energy consumption for the EKG equipment ranged from 0.9 to 4.66 kWh dry ton (Fourie and Jones 2010), when dewatering fine materials such as soft kaolin clay. Less energy consumption can be attributed to these aspects: (1) Short treatment period, and just less than 2 days of operation; (2) periodic (12 h on and 12 h off) power treatment can reduce 54.74% of the total energy consumption compared to continuous treatment (Yuan et al. 2017).

The EKG equipment is suitable for field application. Metals can be directly isolated from the soil matrix via efficient drainage of soil water, which is different to traditional electro-migration and precipitation of metals to the cathode areas (Almeira et al. 2012). Fast dewatering avoids the abundant accumulation of H^+^ and OH^−^ near the electrodes, and thus successfully solved challenges such as anode corrosion and metal precipitation of the cathode. The equipment also combines the electrodes, soil water collection and storage, which overcomes shortcomings such as no facility for collecting water, installation difficulties and maintenance of a large array of electrodes in the field for existed EKG technology (Fourie and Jones 2010). Finally, the operation can be closely related to the crop cultivation process. Saturation of paddy soil with FeCl_3_ and CaCl_2_ will be conducted in the flooding period. Crop planting can normally be carried out after a short period of soil decontamination.

Residuals of externally added Fe and Cl can be reduced to acceptable levels by extending the treatment duration or repeating treatment during the non-growing season. Introduction of FeCl_3_ caused the acidification of paddy soil (Fig. 4), which may be neutralised with lime to adjust the soil pH to initial levels, and reduce the negative impacts of Fe and Cl on crop cultivation. Moreover, iron plaque can block the uptake of Cd by crops and enhance nutrient assimilation. Electro-kinetic remediation using this novel method causes discharge of Cd, Fe, Cl and soil nutrients. The recycling of Fe and Cl, and the recovery of soil nutrients is possible with the following measures: Fe precipitates, if the effluent pH is about 3 (Kaksonen et al. 2014), and then Cd is precipitated in the supernatant at a pH value of beyond 8 (Chen et al. 2017). Iron precipitation and residual Cl can further be used to saturate the paddy soil and dissolve soil Cd before the next electro-kinetic remediation cycle. After removing the effluent Cd, Fe and Cl, the soil nutrients contained in the effluent can be used as fertiliser replacement within the irrigation water to address the soil nutrient loss caused by soil water drainage.

## Conclusions

The proposed EKG equipment is as an effective in situ remediation measure to directly isolate and remove Cd from paddy soil through field plot experiments. Nearly 30% of the total soil Cd content can be separated from the soil matrix through electro-kinetic remediation, which can be regarded as an energy- and time-saving technology for large-scale paddy soil Cd decontamination.

Efficient soil water drainage through gravity and electro-migration promotes the removal of dissolved and mobilised soil Cd after saturation, preventing the occurrence of Cd accumulation and precipitation of Cd on the cathode.

The addition of FeCl_3_ and CaCl_2_ promoted phyto-available Cd liberation from the soil matrix, and led to an acceptable level of Fe and Cl residuals, which can be further reduced by repeated electro-kinetic treatment. The Fe contained in the effluent obtained from the soil water drainage process can be recycled for further soil saturation after separation of harmful metals including Cd.

## Electronic supplementary material

ESM 1 (DOCX 502 kb)
