# Supplementary material for: Improved isolation of cadmium from paddy soil by novel technology based on pore water drainage with graphite-contained electro-kinetic geosynthetics
Source: Environ Sci Pollut Res Int. 2018 Mar 10;25(14):14244–53. doi: 10.1007/s11356-018-1664-4 (PMC5978830; doi:10.1007/s11356-018-1664-4)
Supplement: Supplementary file 1 — (DOCX 502 kb) [file 11356_2018_1664_MOESM1_ESM.docx]

**Supplementary Material**

**Environmental Science and Pollution Research**

**Improved isolation of cadmium from paddy soil by novel technology based on pore water drainage with graphite-contained electro-kinetic geosynthetics**

**Xianqiang Tang^*^ • Qingyun Li • Zhenhua Wang • Yanping Hu •Yuan Hu • Miklas Scholz^**^**

* *Corresponding author after paper publication.* Phone: +86 027 82926192; Fax: +86 027 82926680; E-mail address: [ckyshj@126.com](mailto:ckyshj@126.com) (Xianqiang Tang).

** *Corresponding author before and after paper publication*. Division of Water Resources Engineering, Faculty of Engineering, Lund University, PO Box 118, 22100 Lund, Sweden. Phone: +46 46 222 8920; Fax: +46 46 222 4435; E-mail address: miklas.scholz@tvrl.lth.se (Miklas Scholz).


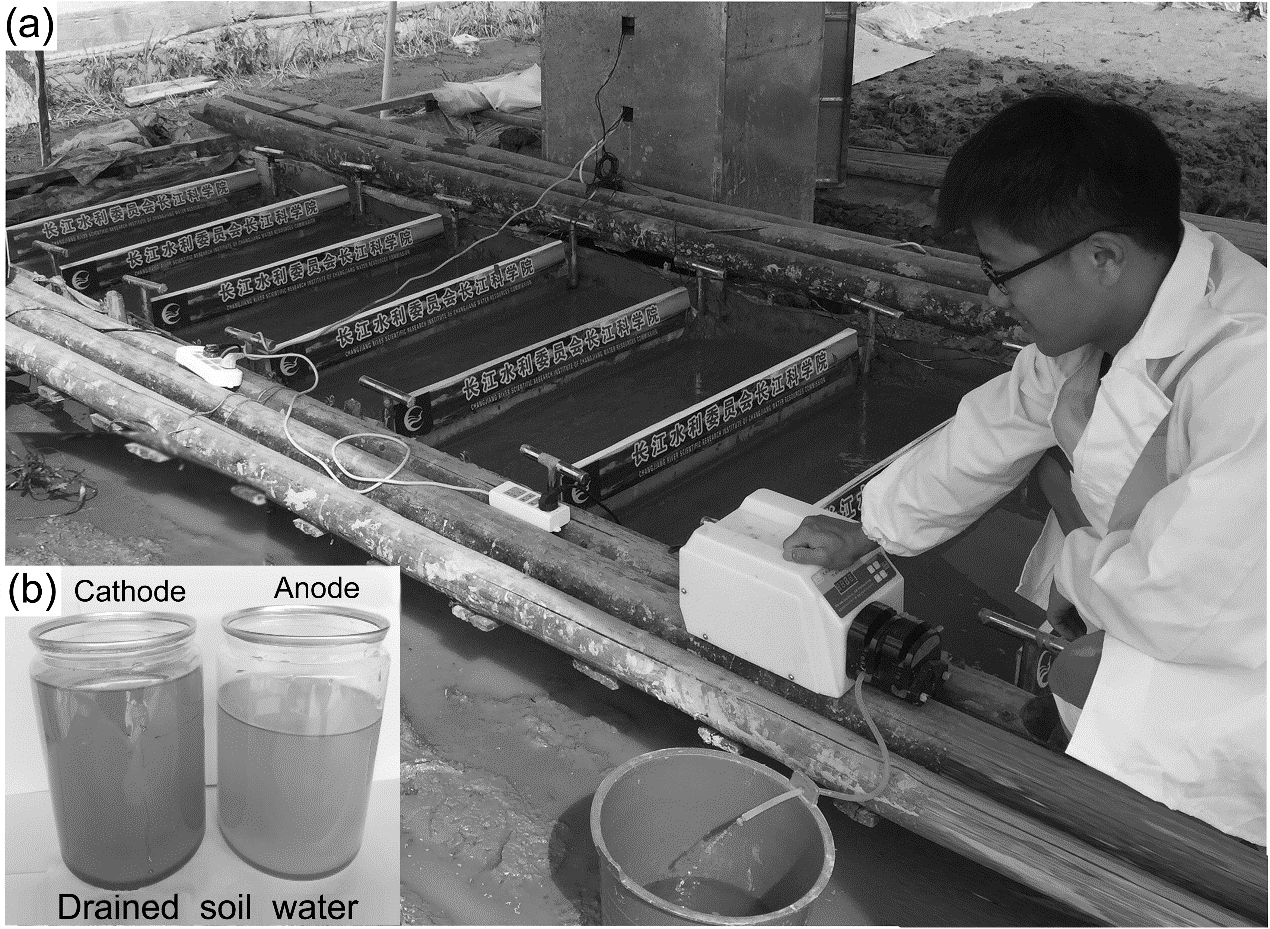


**Figure S1.** (a) Soil water drainage via novel electro-kinetic geosynthetic equipment and effluent collected from both (b) cathode and anode

**Table S1**

Electro-kinetic (EK) remediation experimental design for groups A and B

| Group | Plot dimension (m) | Saturation solution | Electricity applied | EK mode |
| --- | --- | --- | --- | --- |
| A | 1.50×0.86×0.25 | 0.03M ferric chloride + 0.03M calcium chloride | No | None |
| B | 1.50×0.86×0.25 | 0.03M ferric chloride + 0.03M calcium chloride | Yes | 12h On + 12h Off + 8h On |

**Figure S2.** Soil water drainage performance for (a) group A; and (b) group B

**Figure S3.** Variations of electric current intensity measured for group B

**Figure S4.** Variations in anode and cathode effluent iron (Fe) concentrations for (a) group A; and (b) group B

**Figure S5.** Variations in anode and cathode effluent pH for (a) group A; and (b) group B

**Figure S6.** Variations in anode and cathode effluent chlorine (Cl) concentrations for (a) group A; and (b) group B

**Table S2**

Average soil iron (Fe) and chlorine (Cl) residuals for groups A and B

| Group | Fe |  |  |
| --- | --- | --- | --- |
|  | Total added (g) | Removed^a^ (g) | Residual (g) |
| A | 79.80 | 52.34 | 27.46 |
| B | 79.80 | 63.48 | 16.32 |
| Group | Cl |  |  |
|  | Total added (g) | Removed^a^ (g) | Residual (g) |
| A | 252.90 | 165.50 | 87.40 |
| B | 252.90 | 203.91 | 48.99 |

^a^Removal was calculated as the sum content of Fe and Cl contained in the overlying water and soil water drainage.

**Figure S7.** Change in profile distribution of soil iron (Fe) content for (a) group A; and (b) group B with untreated original soil as reference

**Figure S8.** Change in profile distribution of soil chlorine (Cl) content for (a) group A; and (b) group B with untreated original soil as reference
